# Synthesis of 4-Amino-1-Hydroxy-Butane-1,1-Diphosphonate (AHBDP) - Stannous Complexes for the Preparation of Ahbdp-Sn(II)-Tc and its Biodistribution in Rats

**DOI:** 10.1155/MBD.1995.201

**Published:** 1995

**Authors:** Samlee Mankhetkorn, Caroline Blanchot, Muriel Duran-Cordobes, Driss El Manouni, Yves Leroux, Jean-Luc Moretti

**Affiliations:** 1 Université Paris-Nord Laboratoire de Radiopharmacologie de la formation de Recherche Biophysique et Pharmacologie des Biosignaux (DRED) U.F.R. de Santé-Médecine-Biologie Humaine de Bobigny France; 2 Universit Paris-Nord Laboratoire de Chimie Structurale Biomoléculaire U.F.R. de Santé Médecine Biologie Humaine de Bobigny URA 1430 CNRS France

## Abstract

The new potential tracer of bone imaging, AHBDP-Sn(II)-TcO.3H_2_O was synthesized by reducing the TcO_4_^−^  to TcO_2_^+^ in the presence of AHBDP and Sn(ll)’s reducing agent. We found that tin rapidly forms a stable complex with AHBDP, giving AHBDP-Sn(II).3H_2_O. In the excess of AHBDP-Sn(ll).3H_2_O, the AHBDP-Sn(II).3H_2_O coordinates with TcO_2_^+^ to give AHBDP-Sn(II)-TcO.3H_2_O which could polymerise or oligomerise to give hydrophobic species. The overall process appears as a first-order reaction (K= 0.67 ± 0.005s^−1^). In rats, the fixation of AHBDP-Sn(II)-^99m^TcO. 3H_2_O on bone is homogeneous and the scintigraphic images have the same quality as those of 1-hydroxymethane-1,1-diphosphonate-Technetium (HMDP-^99m^Tc). The activity in non-target organs was neglible.


### SYNTHESIS OF 4-AMINO-1-HYDROXY-BUTANE-1,1-DIPHOSPHONATE (AHBDP) - STANNOUS COMPLEXES FOR THE PREPARATION OF AHBDP-Sn(II)-Tc AND ITS BIODISTRIBUTION IN RATS

Samlee Mankhetkorn[1], Caroline Blanchot[1], Muriel Duran-Cordobes[1], Driss El Manouni[2], Yves Leroux[2], and Jean-Luc Moretti[1]

Université Paris-Nord, France
[1] Laboratoire de Radiopharmacologie de la formation de Recherche Biophysique et Pharmacologie des Biosignaux (DRED), U.F.R. de Santé-Médecine-Biologie Humaine de Bobigny,
[2] Laboratoire de Chimie Structurale Biomoléculaire
U.F.R. de Santé, Médecine, Biologie Humaine de Bobigny, URA 1430 CNRS

**Abstract**

The new potential tracer of bone imaging, AHBDP-Sn(II)-TcO.$3H_2O$ was synthesized by reducing the $TcO_4^-$ to $TcO^{2+}$ in the presence of AHBDP and Sn(II)'s reducing agent. We found that tin rapidly forms a stable complex with AHBDP, giving AHBDP-Sn(II).$3H_2O$. In the excess of AHBDP-Sn(II).$3H_2O$, the AHBDP-Sn(II).$3H_2O$ coordinates with $TcO^{2+}$ to give AHBDP-Sn(II)-TcO.$3H_2O$ which could polymerise or oligomerise to give hydrophobic species. The overall process appears as a first-order reaction (k= 0.67 ± 0.005 $s^{-1}$). In rats, the fixation of AHBDP-Sn(II)-$^{99m}$TcO. $3H_2O$ on bone is homogeneous and the scintigraphic images have the same quality as those of 1-hydroxymethane-1,1-diphosphonate-Technetium (HMDP-$^{99m}$Tc). The activity in non-target organs was neglible.

### INTRODUCTION

4-amino-1-hydroxybutane-1,1-diphosphonate (AHBDP) is a diphosphonate derivative. Diphosphonate-technetium complexes such as 1-hydroxymethane-1,1-diphosphonate-technetium (HMDP-Tc), 1-hydroxyethane-1,1-diphosphonate (HEDP-Tc) (Chart 1), etc. are widely used in bone scintigraphy. It is well-known that these complexes can exist as monomers, polymers or oligomers, and also that in technetium, in different oxidation states, frequently as +4 and +5 as well as +3 and +6, different valence states may exist together, depending on the experimental conditions [1-4].

The subsequent reduction of $TcO_4^{(-)}$ from +7 to a lower level of oxidation in the presence of various ligands is essential for radiopharmaceuticals. Stannous chloride ($SnCl_2$) is frequently used as a reducing agent for $TcO_4^{(-)}$. Unfortunately, in aqueous solution, Sn(II) is easily hydrolyzed and oxidized. Its solution is stable only in the presence of hydrochloric acid [5-9]. However, it has been reported that a large excess of $SnCl_2$ in labelling systems affects not only the quality, purity and stability of the radiopharmaceuticals, but also the biological behaviour of $^{99m}$Tc-complexes. Sn(II) forms chelates with many substrates so that the labelling procedures generally yield a mixture of tin and technetium chelates [10-12]. Deutsch proposed that tin can and does bind to Tc complexes [1].

Huigen et al. showed that the HEDP-Sn(II)-Tc has a molecular charge between -4 and -9 at pH 7 and that in an acidic pH , the charge increases [13] .Tji et al showed that the diphosphonate-technetium complexes adsorb on calcium phosphate, as a model of bone adsorption. For the -Tc(III), -Tc(IV), and HEDP-Tc(V) complexes, the adsorption on calcium phosphate increases in the following order Tc(V) < Tc(IV) < Tc(III) [3].

It was observed that the chemical structure of complexes affects the biological behavior. According to its chemical structure, we can predict its biological function. However, the chemical structure of the technetium-complexes was not clearly shown. In this study, we want to report the synthesis and physico-chemical characterisation of the AHBDP-Sn(II)-Tc by using AHBDP-Sn(II).3$H_2O$ as a chelating and reducing agent for the technetium. We have also investigated its biodistribution in rats.

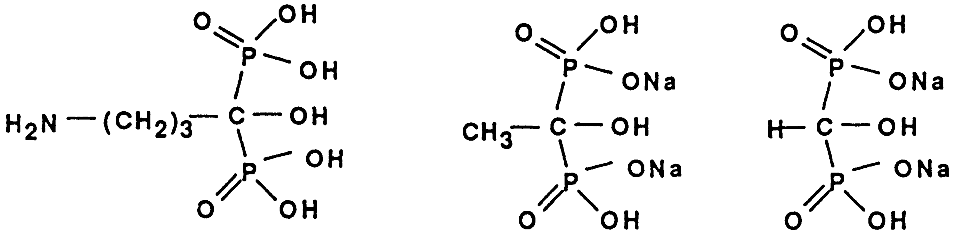

Chart1

### MATERIALS AND METHODS

AHBDP was synthesized following the published procedure[14-15] and its purity was controlled by NMR spectroscopy. Stannous chloride ($SnCl_2$.2 $H_2O$) was obtained from Sigma. Ammonium pertechnetate ($^{99}TcO_4.NH_3$) was obtained from Dupont and sodium pertechnetate ($^{99m}TcO_4$.Na) eluted from the Mo-$^{99m}$Tc generator, was obtained from Cis-Bio Int. HCL normapur grade was obtained from Prolabo . Cocathiline was obtained from Chimika-Biochemika. Whatman paper no. 3 MM was obtained from Bioblock (France). All solutions were prepared with bi-distilled water saturated with nitrogen.

Spectrophotometric measurements were done with a HEWLETT PACKARD 8452 A spectrophotometer and the optical path was 1 cm (URA. CNRS 2056). NMR spectra were recorded, using a NMR JEOL model Fx 100.

Paper chromatograms (methanol : water = 80 :20) was interpreted with a Chromelec Nu-102 radiochromatogram.

The AHBDP is dissolved in water and its solution is stable for many hours.

$SnCl_2$ was dissolved in a 0,1 N HCl solution. The dissociated constant, $k_a$, is equal to 5.7 $10^{-3}$ $M^{-1}.s^{-1}$ [6].The Sn(II) concentration is measured spectrophotometrically by complexation with cocathiline, using $\varepsilon_{523}$ = 2773 $M^{-1}.cm^{-1}$.

The $^{99}TcO_4^{(-)}$ concentration is determined spectrophotometrically, using $\varepsilon_{248}$ = 6220 $M^{-1}.cm^{-1}$.

Potentiometric titrations were done with a CG-837 pH meter at 22°C, using the Geraté Schott glass electrode , model N 52A. A solution (25 ml) containing 0:1, 1:1 and 1:3 metal to ligand molar ratios was introduced into the titration cell, and the proton ion concentration was determined by a number of successive readings after addition of small quantity of standard 0.01 N NaOH.

The tissue distribution was performed at 30 minutes, 1, 2 and 3 hours post injection of 74 MBq per rat. After dissection of rat, organs sample activities were counted by NaI(Tl) scintillator (Packard COBRA 5010).

### RESULTS

I. Complexation of AHBDP-Sn(II)

The kinetic studies and final product analysis were performed for several concentration ratios of AHBDP and Sn(II). The pH value of the solutions varied between 1 to 11.

I.1. Spectrophotometric studies:

The initial AHBDP and Sn(II) concentrations varied from $10^{-4}$ to $10^{-2}$ M. The solution of AHBDP did not absorb UV-Vis light. At pH 3, the mixture solution of AHBDP and Sn(II) gave an absorption band at 224 nm. In alkaline solution, the absorbance at this wavelength decreased while a new band appeared at 276 nm. The solutions were stable for less than 5 hours.The reaction was done in less than 1 minute.

For the study of the complexes, two series of experiments were performed. In one series, the concentration of AHBDP was varied in the presence of a constant excess of Sn(II) ([Sn(II)] $=10^{-2}$ M). However, in the other, the Sn(II) concentration was variable and the AHBDP constant; [AHBDP] = $10^{-2}$ and 5 x $10^{-2}$ M. The pH of the solutions was adjusted to 3, using 1.0 N HCl. The formula of the complexes was further investigated by spectrophotometrical measurements[16]. The absorption band at 224 nm increased as the molar ratios of Sn(II):AHBDP increased. This absorbance was stable when the molar ratio was equal to 1. In these conditions, the reaction was done with stoichiometry 1:1.

I.2. Potentiometric titrations of AHBDP-Sn(II)

The titration curves are illustrated in Figure 1 for AHBDP chelate of Sn(II) at 1:0, 1:1 and 1:3 ratios of metal to ligand. There is evidence of complex formation between AHBDP and Sn(II). Titrations of equimolar amounts of AHBDP and Sn(II) resulted in a steep rise at [NaOH] = 0.002 M, corresponding to the formation of a 1:1 complex. A 1:3 molar ratio resulted in steep rise at [NaOH] = 0.0014 M. This indicates that AHBDP-Sn(II) exists as 1:1 complex.

In purified water, AHBDP is present in the zwitterionic form. The $pK_a$ of AHBDP were determined, $pk_{a1}$, $pk_{a2}$ and $pk_{a3}$ are equal to 3, 6.8 and 10.

In order to identify the products of the reaction, two series of experiments were carried out in the following conditions: [AHBDP] = [Sn(II)] $=10^{-2}$ M. In the first series the pH was equal to 2, while in the second, it was adjusted to 11 with NaOH. After the completion of the reaction, the solutions were lyophilized and the products were analyzed by $^{31}$P-NMR. It should be noted that the lyophilized powder at pH 11 was instable in the ambient conditions; its color changed from white to black and became insoluble in water. On the other hand, the lyophilized powder at pH 2 was stable and soluble in water. The $^{31}$P- NMR spectrum was very different from the one that obtained from the pure AHBDP (The pure AHBDP $\delta = 19.5$ ppm; In complex solutions this peak has disappeared). This indicated that there is a modified electronic environment of phosphorus and all of AHBDP is complexed.

2. Complexation of AHBDP-Sn(II)-Tc

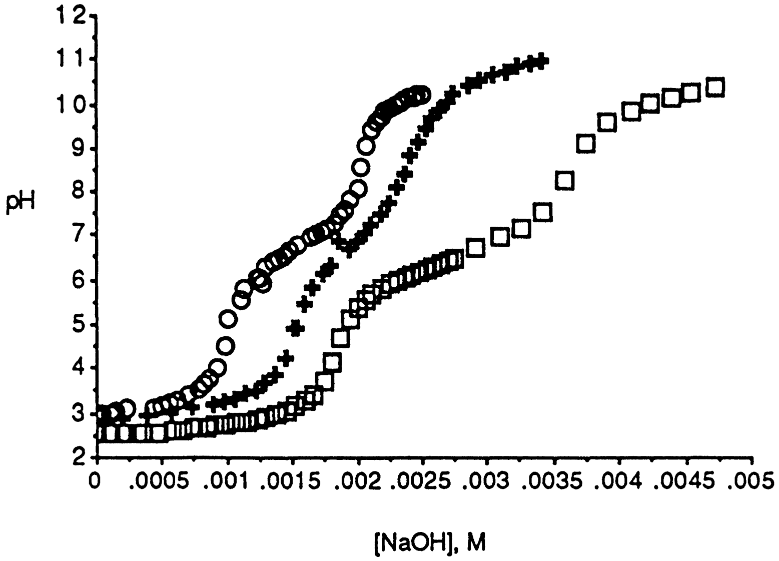

Figure 1: Potentiometric titrations of AHBDP-Sn(II) for (o) 1:0, (□) 1:1, (+) 1:3 molar ratios of metal ligand, [AHBDP] = $10^{-3}$ M, [NaOH] = 0.1 N.

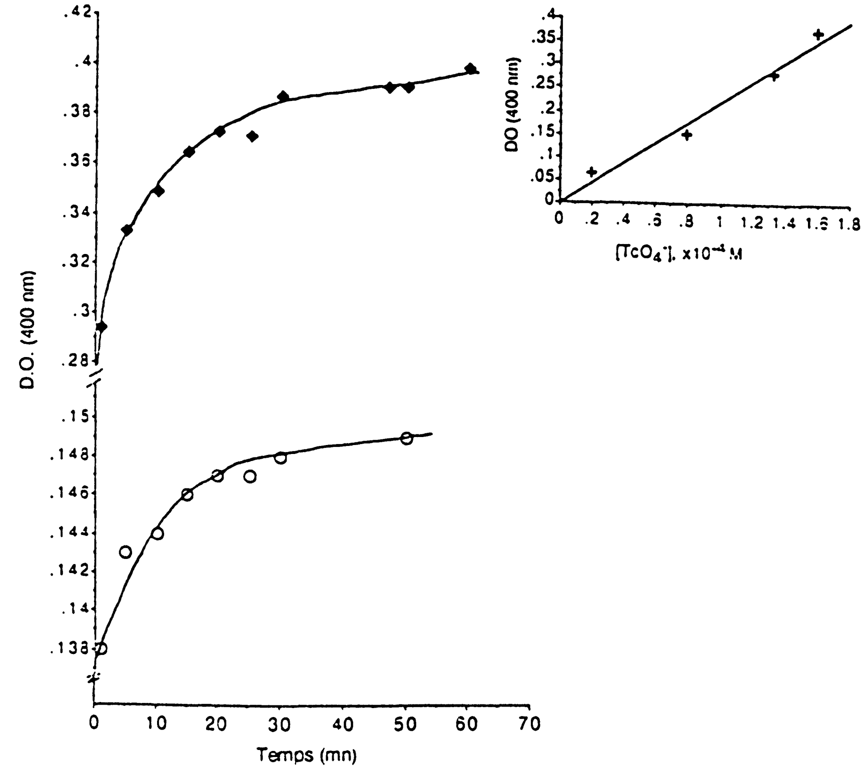

Fig. 2: Complexation of AHBDP-Sn(II) by Tc in the excess solution of AHBDP-Sn(II), [AHBDP-Sn(II)] = $10^{-3}$ M, pH 3; (o) $[TcO_4^-]$ = 7.9 x $10^{-5}$ M and (♦) $[TcO_4^-]$ = 1.6 x $10^{-4}$ M. Computed monoexponential

Inset: Variation of absorbance at 400 nm, at plateau level, of AHBDP-Sn(II) chelates Tc, in function of $[TcO_4^-]$. Line is "linear least-squares fit" of data.curves (points are experimental).

We have verified that AHBDP-Sn(II) solutions were stable for several hours and we always used fresh solutions in our experiments.

2.1. Spectrophotometric studies

In this series of experiments, the concentration of AHBDP-Sn(II) varie from $10^{-6}$ to $2 \times 10^{-3}$ M and the concentration of $TcO_4^{(-)}$ varied from $10^{-6}$ to $10^{-2}$ M. The pH varied from 1.5 to 11.

Immediately after the mixture of AHBDP-Sn(II) and $TcO_4^{(-)}$, the solution became yellow. For the following series of solutions with equimolar amounts of AHBDP-Sn(II) and $TcO_4^{(-)}$ and the excess of the $TcO_4^{(-)}$, the solutions became opaque and were composed of a brown precipitate.

For the excess solutions of AHBDP-Sn(II), [AHBDP-Sn(II)] > $10[TcO_4^{(-)}]$, the evolution of the absorption spectra was investigated. The absorbance at 224 nm immediately increased after the addition of $TcO_4^{(-)}$ and then decreased before stabilising. There was a new absorption band at 400 nm. The evolutions of the absorbance at 400 nm are reported in figure 2. A pseudo plateau was reached by absorbance, within 25 minutes; however, the absorption spectra changed slowly and a yellow precipitate appeared over 72 hours. It should be noted that the pseudo plateau was attained independently of the initial concentrations of the reagent, as it can be seen in the inset of Figure 2 . In these conditions of stoichiometry 1:1 , the composition of the solution at the pseudo plateau was thus independent of the reagent concentrations and the average extinction for the complexes could be calculated using the slope of the straight line of inset of Figure 2 , $\varepsilon = 2190\ M^{-1}.cm^{-1}$. All curves, such as the one in figure 2, could be analysed according to a first-order kinetic law, with a rate constant of $0.67 \pm 0.005\ s^{-1}$.

We have verified that the formation of the complex was independent of pH and that complex was stable as a function of dilution.

2.2 Potentiometric titrations of AHBDP-Sn(II)-Tc

The titration curves are illustrated in figure 3 for AHBDP-Sn(II) chelates Tc for 1:0, 1:10 and 1:25 ratios of metal to ligand. In steep inflection, the three curves were identical, there were no protons released from the complex for pH < 6. It is possible that after the reduction of the $TcO_4^{(-)}$ to $TcO^{(2+)}$, the AHBDP-Sn(II) chelates $TcO^{(2+)}$ forming a complex as shown in scheme 2. The 5 could either polymerise or oligomerise to give the formation of the hydrophobic species, indicated by the yellow precipitate in the solution after 72 hours.

3. Biodistribution of AHBDP-Sn(II)-$^{99m}$Tc in rat

3.1 Preparation of the AHBDP-Sn(II)-$^{99m}$Tc

Paper chromatography of AHBDP-Sn(II)-$^{99m}$Tc (prepared under the conditions described above) showed that all the activity was localised at the origin. These results showed that the AHBDP-Sn(II)-$^{99m}$Tc was almost free from $Na^{99m}TcO_4$. The yield of labelling was about 96 % and the complexes was stable for at least two hours.

3.2. Tissue distribution

Uptake of the AHBDP in various organs of rats at 0.5,1, 2 and 3 hours post injection, indicated that the greater part of the complex was distributed in the rats' bones, kidneys and urinary bladders. Lungs, spleen, liver, heart and muscle showed negligible activity. Per gram organ uptake, as shown in table 1, was found to be highest in bone as compared to the other organs.

Studies on blood clearance of the AHBDP showed that during the initial post injection period, there was a rapid loss of activity in the blood and it was reduced to only 6% of the injection dose within 30 minutes. Thereafter, further clearance was lower at 1 hour post injection, the blood still showed 2% of the injected activity.

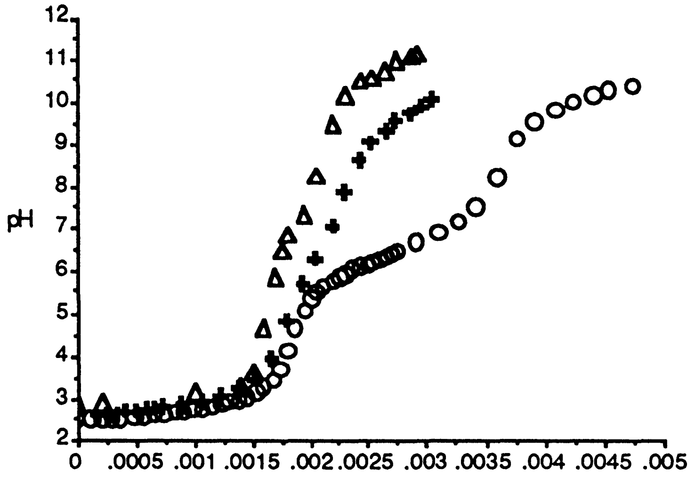

Fig. 3: Potentiometric titrations of AHBDP-Sn(II)-Tc for (o) 1:0, (+) 1:10 and (Δ) 1:25 molar ratios of metal ligand, [AHBDP-Sn(II)] = $10^{-3}$ M, [NaOH] = 0.1 N.

| **Organs** | 30 min | | 1 hour | | 2 hours | | 3 hours | |
|---|---|---|---|---|---|---|---|---|
| | HMDP | AHBDP | HMDP | AHBDP | HMDP | AHBDP | HMDP | AHBDP |
| tibia | 44 | 29 | 41 | 29.7 | 41.5 | 46 | 49 | 43 |
| vertebra | 39 | 33 | 17 | 11.9 | 20 | 21 | 22 | 20.8 |
| lt. kidney | 0.9 | 3.2 | 0.3 | 1.43 | 0.23 | 0.7 | 0.4 | 0.64 |
| rt. kidney | 1 | 3.1 | 0.22 | 1 | 0.22 | 1 | 0.3 | 0.6 |
| liver | 0.5 | 1.6 | 0.33 | 0.5 | 0.4 | 0.5 | 0.5 | 0.4 |
| spleen | 0.04 | 0.3 | 0.06 | 0.2 | 0.04 | 0.4 | 0.05 | 0.25 |
| lungs | 0.12 | 1.4 | 0.04 | 0.38 | 0.04 | 0.1 | 0.04 | 0.15 |
| heart | 0.05 | 0.13 | 0.01 | 0.04 | 0.01 | 0.03 | 0.01 | 0.03 |
| muscle | 5 | 13 | 1.05 | 4.6 | 5 | 3.96 | 1.6 | 2.1 |
| blood | 3 | 6.5 | 0.5 | 2.1 | 0.3 | 2.8 | 0.3 | 2.14 |

Table 1; Accumulated activities in different organs of rats of the AHBDP and the HMDP at various time post injection of 74 MBq per rat. The rats were dissected and organ sample activities were counted by gamma countor. The activities were calculated according to the following equation, (Act./Org.) x (rat wt./DI).

### DISCUSSION

1. Complexation of AHBDP-Sn(II)

The experimental data indicated that Sn(II) has formed a stable 1:1 complex with AHBDP. Its oxidation state is +2. This is in agreement with the previous work performed by Nakayama et al[11-12]. We propose, for the overall reaction, the mechanism shown in scheme 1, at pH 3, where the AHBDP is in the predominating species 2, and the Sn(II) is solvated. The first step could be the exchanging of $H_2O$ by oxygen anions leading to the hydrated complexes . Next, the coordination of Sn(II) is stabilized by the atom donor, nitrogen atom of the amine group, with the elimination of one molecule of water, corresponding to 4.

2. Complexation of AHBDP-Sn(II)-$^{99}$Tc

The study of spectrophotometric and potentiometric titrations shows that the technetium has formed a complex, chelated by AHBDP-Sn(II).3$H_2O$ with hydroxyl groups, giving AHBDP-Sn(II)-TcO.3$H_2O$. This is in agreement with the previous works reported by Tji et al. and Deutsch et al.. It was observed that for equimolar amounts of AHBDP-Sn(II) and $TcO_4^{(-)}$ and the excess of the $TcO_4^{(-)}$, the solutions are almost formed with the brown precipitated (as mentioned above) and we propose that the technetium remains at the degree of oxidation +4. The Tc(IV) formed the complex with AHBDP-Sn(II).2$H_2O$ and/or hydrolyzed to give $TcO_2$.2$H_2O$ that precipitated. For the excess of ligand, the reaction was done in several steps. The first step was the reduction of $TcO_4^{(-)}$to $TcO^{(2+)}$ by AHBDP-Sn(II).2$H_2O$. The second step was the chelation of $TcO^{(2+)}$ by BHADP-Sn(II).2$H_2O$ that we propose the mechanism in scheme 2. The first step of the reaction was the exchange of two molecules of water by the hydroxyl function of the phosphonic groups, including the addition of the atom donor, Sn(II) to Tc, giving the hydrated complexes. The product had intramolecular rearrangement, with the elimination of one molecule of water to give 5 The second step was the polymerization or oligomerization of the 5 to give the hydrophobic species. Either the first or the second step of the mechanism could have given the constant rate of $0.67 \pm 0.005\ s^{-1}$.

3. Biodistribution

It is clear that AHBDP-Sn(II)-$^{99m}$TcO.3$H_2O$ and HMDP-$^{99m}$Tc has a high affinity to bone. The kinetics and saturation time of the uptake to bone was not significantly different in either complex. AHBDP-Sn(II)-$^{99m}$TcO.3$H_2O$ is eliminated more rapidly than HMDP-$^{99m}$Tc. The signal / background ratio of AHBDP-Sn(II)-$^{99m}$TcO.3$H_2O$ was lower than that of HMDP-$^{99m}$Tc, assessed by tibia and vertibra versus blood (figure 4) and muscle (Figure 5). Accumulated activities in non-target organs was negligible. The biological behavior of AHBDP-Sn(II)-$^{99m}$TcO.3$H_2O$ was closed to other diphosphonate compounds; HMDP,HEDP, etc. We found that the AHBDP-Sn(II)-TcO. 3$H_2O$ complex was stable , with the variation of pH and dilution. In rats, it fixed homogeneously on bone and the scintigraphic images had the same quality as that of HMDP-$^{99m}$Tc image. We propose to consider the AHBDP-Sn(II)-$^{99m}$TcO.3$H_2O$ as a good tracer for bone imaging.

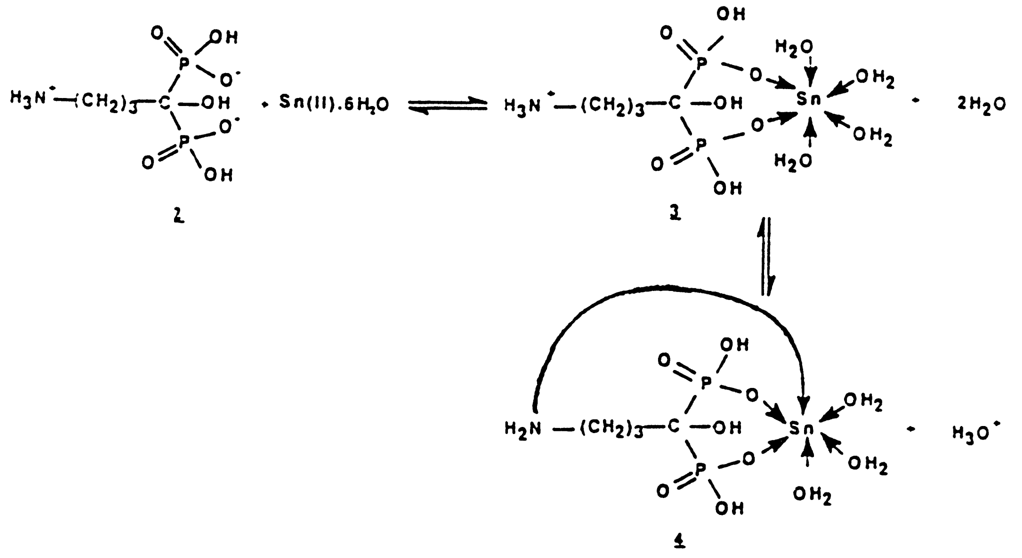

Scheme1

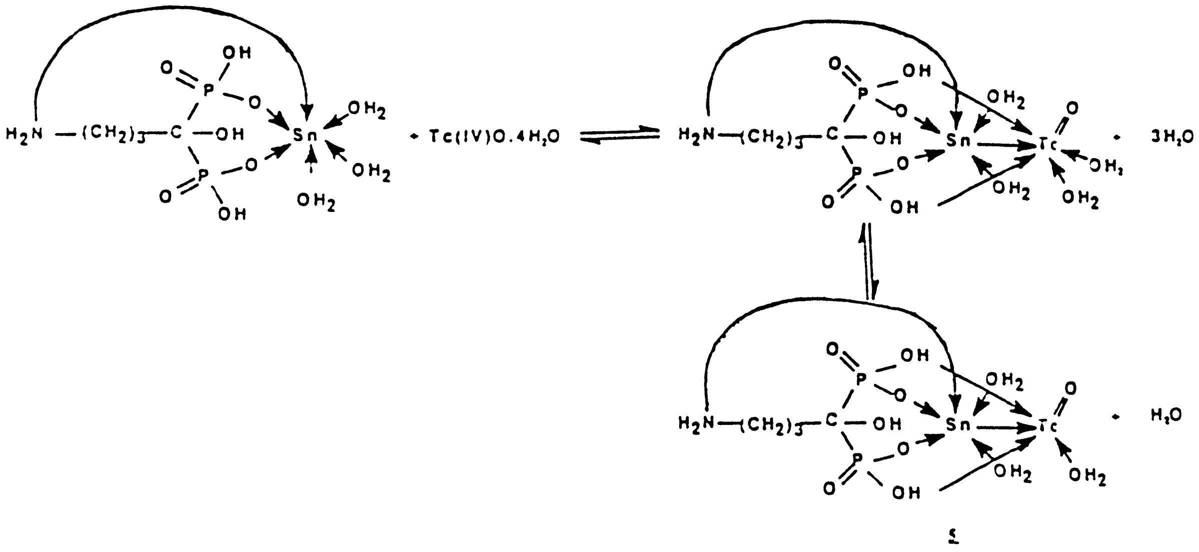

Scheme2

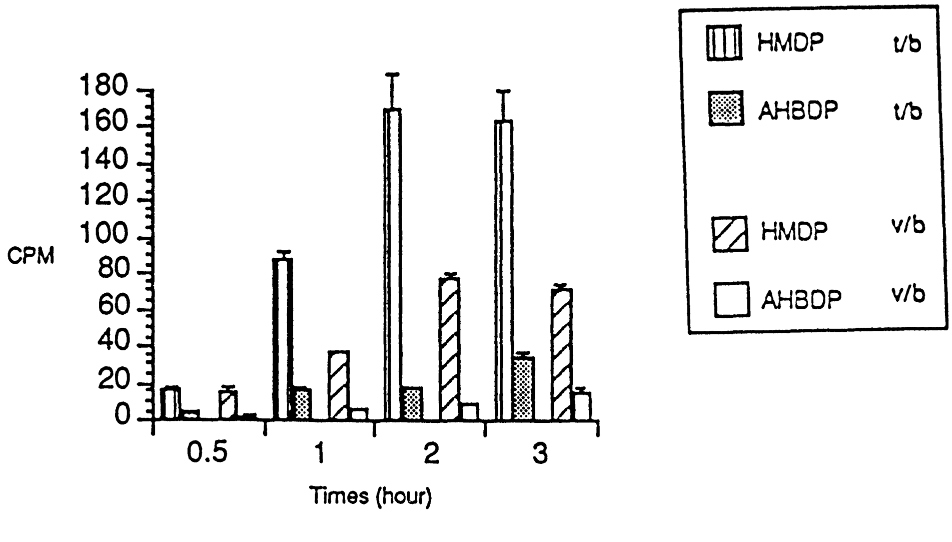

Fig.4: Variation of the ratio of the activities of bone related to the activities in blood, data form table 1.

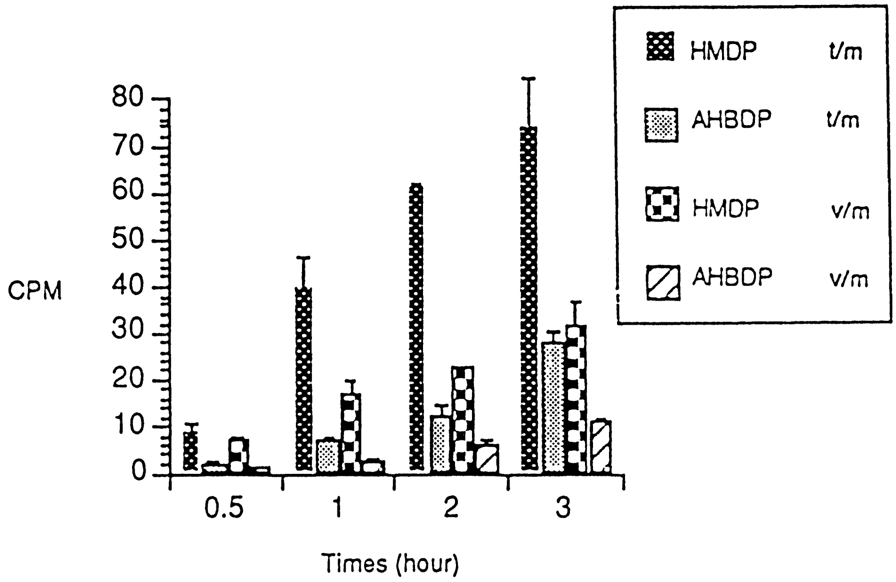

Fig. 5: Variation of the ratio of the activities of bone related to the activities in muscle, data form table 1.

Acknowledgements: The authors would like to thank to Pr. A. Garnier-Suillerot and Dr. M. Fiallo for critical reading of the paper. Thanks are due to Dr. C. Dufau for recording $^{31}$P-NMR. S. MANKHETKORN is grateful for financial support from the Thai Government.

### REFERENCES

1. Peacock R.D., Schwochau K., Seidel A., Stadl auer E., *Gmelin Handbook of Inorganic Chemistry, Tc Supplement.* vol.1, 8 th ed.: Springer-Verlag Berlin.Heidelberg, 1982: 271-333.
2. Nieuwland R.J.A., Das H.A., de Ligny C.L.. *Appl. Radiat. Isot.* 1989; 40 (.2): 153-157.
3. Tji T.G., Vink H.A., Gelsema W.J. , de Ligny C.L..*Appl. Radiat. Isot.* 1990; 41 (1): 17-28.
4. Le Tuang Minh, Lengyel T..*Radioanal. Nucl.Chem, Letters 128*,1988; 5: 417-422.

5. de Alleluia I.B., Burgess J., Peacock R.D., Schwochau K., *Gmelin Handbook of Inorganic Chemistry, Tc Supplement,* **vol.2, 8 th ed.** :Springer-Verlag Berlin.Heidelberg, 1982: 153-242.
6. Bévillard P., Faivre R., Godfrin A., Lemanceau B., Pascal P., Tchakirian M., Weiss R., *Nouveau traité de chimie minérale, Germanium, étain and plomb,* **vol. 8**: Masson et Cie, Etideurs, Paris, 1963: 312-20.
7. Ikda I., Inoue O., Kkurata K.. *Appl. Radiat. Isot* 1976; **27**: 681-688.
8. Srivastava S.C., Meinken G., Smith T.D., Rich ards P.. *Appl. Radiat. Isot,* 1977; **28 (1)**: 83-95.
9. Bumbalova A., Haranek E., Harangozo M., Krenek P., Butvin P.. *Radioanal. Nucl.Chem, Letters 137,*1989; **4**: 251-257.
10. Faiz-ur-Rehman, Shamas-uz-Zaman, Shahid M.A., Imran S.L., Ashraf M., Waheed Akhtar M.. *Appl. Radiat. Isot,* 1986; **37 (3)**:.249-255.
11. Nakayama M., Terahara T., Wada M., Harada K., Sugii A., Egawa H.. *App.Pol.Sc.,*1991,**43**; 2231-2236.
12. Nakayama M., Terahara T., Wada M., et al. *Nucl. Med. Commun,* 1991; **12**:147
13. Huigen Y.M., Diender M., Gelsema W.J., de LignyC.L.. *Appl. Radiat. Isot,* 1991; **42 (1)**: 71-76.
14. El Manouni D., Leroux Y., Burgada R..*Phosphorus and sulphur,* 1989; **42**: 73-83.
15. Tromelin A., El Manouni D., Burgada R.. *Phosphorus and sulphur,* 1986, **27**, 301-312.
16. Havey A.E., Manning J.R., Manning D.L.. *Contribution form the chemistry department of University of Louisville Oct.1950*; **72**: 4488-4493.

**Received: January 1, 1995 - Accepted: February 13, 1995 - Received camera-ready revised version: May 12, 1995**

